# Motivated Cognition in Cooperation

**DOI:** 10.1177/17456916231193990

**Published:** 2023-10-26

**Authors:** Susann Fiedler, Hooman Habibnia, Alina Fahrenwaldt, Rima-Maria Rahal

**Affiliations:** 1 Vienna University of Economics and Business, Austria; 2 Max Planck Institute for Research on Collective Goods, Bonn, Germany; 3 Faculty of Human Sciences, University of Cologne, Germany

**Keywords:** motivated cognition, belief updating, cooperation, information search

## Abstract

Successful cooperation is tightly linked to individuals’ beliefs about their interaction partners, the decision setting, and existing norms, perceptions, and values. This article reviews and integrates findings from judgment and decision-making, social and cognitive psychology, political science, and economics, developing a systematic overview of the mechanisms underlying motivated cognition in cooperation. We elaborate on how theories and concepts related to motivated cognition developed in various disciplines define the concept and describe its functionality. We explain why beliefs play such an essential role in cooperation, how they can be distorted, and how this fosters or harms cooperation. We also highlight how individual differences and situational factors change the propensity to engage in motivated cognition. In the form of a construct map, we provide a visualization of the theoretical and empirical knowledge structure regarding the role of motivated cognition, including its many interdependencies, feedback loops, and moderating influences. We conclude with a brief suggestion for a future research agenda based on this compiled evidence.

Humanity faces many issues that can only be overcome through collective effort and cooperation. As the scale of these issues increases beyond the local to a global level, we also need to scale up our collectively coordinated cooperative action (Dawes, 1980) to address challenges such as the climate crisis, viral pandemics, antimicrobial resistance, and unequal access to wealth and to opportunities in life. The choice to cooperate is rooted deeply in personal preferences (e.g., social preferences) and beliefs about others’ future behavior (Beckes & Simpson, 2012). Cooperative choices are swayed by social norms, which shape our beliefs of others’ behavior and, often, set expectations for our own actions (Balliet & Van Lange, 2013; Kocher et al., 2015; Pletzer et al., 2018). However, individuals tend to form self-serving beliefs of themselves, of others, and of the decision situation, including the applicable social norm, which influence their cooperation behavior (Foerster & van der Weele, 2021). Consequently, such motivated beliefs pose a threat to effective cooperation.

Whereas in the past cooperation tended to take place in groups of manageable sizes with many face-to-face interactions and similar linguistic, cultural, and religious backgrounds, many real-world cooperation settings are now defined by sparse connectivity, in which individuals act independently in settings of high anonymity with little or no direct communication. Such sparsely connected social environments make it more difficult for decision makers to efficiently learn about a population’s underlying distribution of norms and preferences. In addition to this social ambiguity, uncertainty also exists concerning the environment in which the decision is set (e.g., when decision makers do not know the size of the public good or its provision point; Wit & Wilke, 1998). To add even more complexity, cooperation problems often require some extent of individual sacrifice, in which a failure to endure such sacrifice is often associated with a failure of moral character, making cooperation a very personal matter. Given high social and environmental uncertainty and high potential costs for the self and social image, it is no surprise that the process of forming and updating beliefs about potential cooperation is vulnerable to distortion, with trickle-down consequences for cooperative behavior. For example, such distortions could lead actors to overestimate available resources (e.g., *big pool illusion*; Gärling et al., 2002; Messick & McClelland, 1983) or the level of existing risks (Akerlof & Dickens, 1982), leading to uncooperative behavior misaligned with the actual decision setting.

These belief distortions are typically systematic and influenced by preexisting attitudes and prior beliefs. In this article, we examine the role of motivated cognition, that is, the mental processes of information search and processing by which systematically distorted beliefs are formed and updated in the context of cooperation. Our starting point is a summary of findings on the general relationship between cognition and cooperation, followed by a comprehensive review of the literature on motivated cognition. We then outline the role of beliefs (individuals’ mental constructs about others or their operational environment), examining their relative stability and capacity for change. Finally, we explore the processes of motivated cognition in human cooperation, integrating relevant literature and creating a visual construct map that organizes current knowledge (Fig. 1; Gray, 2017). This visual framework accentuates individual studies’ theoretical contributions while illustrating their interconnectedness. Our exploration includes insights into individual differences and situational factors shaping motivated cognition. We discuss the map’s components throughout the article, using it as a guide through the existing literature.

**Fig. 1.**
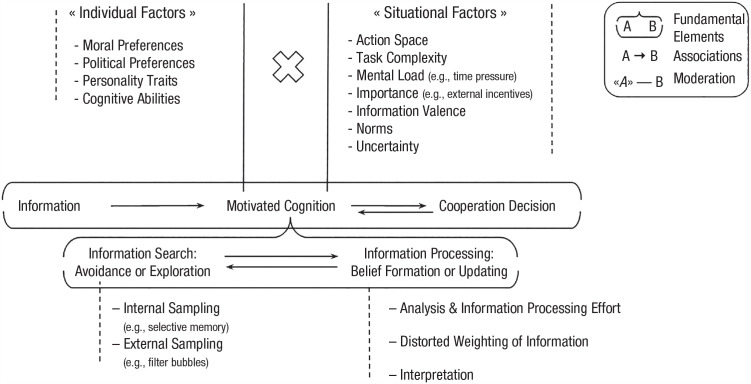
Concept map of the role of motivated cognition in cooperation.

## Cognition and Cooperation

When we decide whether to help others or to pool joint resources to achieve a cooperative goal, what we believe about others’ (beliefs about) cooperative behavior plays an enormous role in determining our actions. Our cooperation decisions are often guided by our perceptions of the social norms governing the group we belong to. These perceptions include the social appropriateness others ascribe to a specific action, others’ likelihood of performing this action themselves, and their willingness to enforce relevant norms via interventions or punishment (Bicchieri, 2005; Fehr & Fischbacher, 2004). For example, in the context of social dilemmas, in which collective interest competes with self-interest (Dawes, 1980), the beliefs decision makers hold about others’ cooperativeness are one driving factor of welfare-maximizing cooperation. Believing that others will cooperate can motivate cooperation (Castro Santa et al., 2018). Conversely, holding beliefs about unfairness in competitive contexts or being able to construct unethicality as permissible can lead individuals to act selfishly (Bersoff, 1999; Hansson et al., 2021). Beliefs, in this context, can assume various forms, such as group stereotypes or self-conceptualizations. These mental constructs significantly influence mental-state inference and, by extension, cooperative behavior. For instance, Ames (2004a, 2004b) demonstrated that when observers perceive themselves to be similar to the target, they tend to rely less on stereotyping and lean more heavily on social projection. Conversely, when they perceive themselves to be different from the target, they often disregard social projection, resorting instead to stereotypes as a basis for judgment. These dynamics significantly influence outcomes in social-dilemma games such as the classic prisoner’s dilemma and can result in cooperative as well as competitive behavior (Ames et al., 2012).

However, research shows that the relationship between beliefs (whether biased or not) and cooperative behavior can strongly vary on the basis of individual preferences and attitudes. In economic lab studies, roughly 50% of participants are labeled conditional cooperators, flexibly basing their cooperation decision on their construction of the current situational and social demands (Bicchieri et al., 2022; Fischbacher & Gächter, 2006; Fischbacher et al., 2001). The remainder are categorized either as individualistic actors (approximately 30% of participants prioritize their own outcomes without consideration for others; Messick & McClintock, 1968) or, infrequently, as pure altruists (those who optimize others’ outcomes without self-regard). The latter either presuppose others’ altruism (false-consensus effect; Blanco et al., 2014) or they do not tie their actions to their beliefs about others’ cooperativeness at all. This group may connect their actions more closely to their self-perceptions, leading some scholars to propose that designating prosocial “roles” to group members even in childhood, such as “the helper,” could be a tactic to bolster group cooperation (Von Hippel & Trivers, 2011).

Crucially, it has been argued that individuals frequently misperceive a population’s level of cooperation willingness (Aoyagi et al., 2021; Santos et al., 2021), often in line with their aforementioned cooperation type. For example, according to a meta-analysis by Pletzer et al. (2018), prosocial individuals aiming to advance both their own and others’ outcomes expect more cooperation from others in social dilemmas than individualistic individuals. These results may be explained by the pivotal role of trust (i.e., the expectation of others’ benevolent intentions) in fostering cooperation, especially in situations with high conflicts of interest (Balliet & Van Lange, 2013). In other words, those intrinsically motivated to cooperate may skew their perception of others’ cooperativeness to be better able to trust them (and thus feel comfortable cooperating).

These results suggest that a deeper investigation of the role of motivated cognition in cooperation could help to understand when and why individuals cooperate and to design supporting interventions. As a first step in unraveling this link between the processes of motivated cognition and cooperation, we construct a synthesis of the various theories and conceptualizations of motivated cognition itself.

## Theories and Conceptualizations of Motivated Cognition

How beliefs are formed and updated is the subject of a wealth of studies in the fields of social and cognitive psychology as well as (behavioral) economics, both regarding individual (Carver et al., 2010; Puri & Robinson, 2007; Weinstein & Klein, 1995) and group (Anand et al., 2004; Schrand & Zechman, 2012) decision-making. Standard economic theory assumes individuals utilize all necessary information before deciding (Stigler, 1961). In this tradition, belief-updating processes are often modeled as a form of Bayesian updating (Bennett, 2015): Agents weigh incoming information against the strength of their priors, regardless of their fit with prior beliefs and goals. In contrast to this standard economic notion, early psychological theorizing posited that individuals feel discomfort when perceiving information contradictory to prior beliefs (cognitive-dissonance theory; Festinger, 1957). The resulting feeling of dissonance is then assumed to motivate individuals to engage in dissonance-reduction strategies (e.g., by changing, justifying, and denying behavior or cognition) as well as to actively avoid situations and information likely to trigger dissonance (Festinger, 1957). In 1980, psychologists termed this biased mechanism “motivated belief updating and reasoning” (Kruglanski & Freund, 1983; Kunda, 1987). Empirical evidence supports the idea that parts of the Bayesian updating processes are not rational but motivated by initial preferences and attitudes (Druckman & McGrath, 2019). Further research advanced cognitive-dissonance theory by distinguishing between accuracy-directed and directionally directed motivated cognition (Kunda, 1990): Whether individuals are motivated to be accurate or willing to arrive at a predetermined conclusion about the world, motivated cognition affects their belief formation and updating through cognitive processes. Building on these ideas, Lodge and Taber’s (2000, 2005) three-component model for motivated cognition emphasized the role of affect for motivated cognition, suggesting that social concepts are tagged with negative or positive affect in memory. Consequently, partisan goals and further information processing are driven by automatic affective processes that predict biased beliefs’ direction and strength.

The importance of affect for motivated cognition was also met with research interest in political science. In *The American Voter*, Campbell et al. (1960) discussed the idea that individuals form emotional connections with their beliefs, hindering them from aligning their political preferences accurately. Party identification is argued to create a filter through which individuals perceive information that aligns with their partisan orientation. The strength of this party affiliation intensifies the selection process and leads to perceptual distortion.

Taken together, much research in this field takes a deficit perspective by presenting motivated cognition as an irrational behavioral bias. However, ignorance of information or the distortion of beliefs might be effective strategies depending on the respective environment. For example, Gigerenzer and Brighton (2009) compiled studies indicating that ignoring information may reduce excessive complexity of information processing and thus result not only in faster inferences but also more accurate ones, especially in situations characterized by high informational complexity and uncertainty. Ignorance can even be seen as a successful cognitive strategy using the representation and structure of information in an environment to make good decisions (i.e., decisions maximizing net benefits matching the agent’s preferences; Todd & Gigerenzer, 2012).

In economics, motivated cognition gained traction after Akerlof and Dickens (1982) integrated cognitive-dissonance theory into the standard rational-choice framework. This integration postulated that individuals have preferences not only about the state of the world but also about their beliefs. The authors assumed that each individual “has a psychic cost of fear” (p. 311) that can be reduced by holding certain beliefs (e.g., about the safety of a job). Second, individuals can control their views and manipulate their beliefs by selecting information confirming their desired beliefs. With this extension, the authors allowed economic theory to derive more psychologically realistic assumptions and open the field to integrating deviations of rational beliefs into economic modeling. Following in these footsteps, Bénabou (2015) and Bénabou and Tirole (2002, 2004, 2016) proposed a unified framework to capture concepts such as overconfidence, information avoidance, wishful blindness, overoptimism, and groupthink under the umbrella term “motivated cognition.” In this sequential model, agents at different stages may choose actions or perceive signals diverging from Bayes’s rules, resulting in a distorted belief. Such motivated cognition is considered self-deception, which can be about the self (e.g., morality, intelligence, identity) or the environment (e.g., trust in other individuals, religion, ideology) and aims at fulfilling psychological and functional needs (Bénabou, 2015). Overall, economic theories depict belief distortions as a result of individuals being motivated by self-interest. Still, this view extends beyond covering only payoff maximization to include self- and social-image concerns and preferences.

Current research relates the phenomenon of motivated cognition to the widely known crowding-out effect in economic markets, referring to it as “crowding-out in attention” (Epley & Gilovich, 2016). Hidden goals, such as protecting one’s self-image or group identity, can guide attention and cognition in a way that seems irrational from an outside perspective.

Integrating these ideas of motivated cognition into the context of the law, Kahan and colleagues (2012) introduced the concept of identity-protective cognition. This account posits that individuals form and maintain beliefs that signal their loyalty to their in-group. In contrast to the work in psychology, political science, and parts of economics introduced above, Kahan et al. proposed that this mechanism is often deliberate and focused on strategic alignment within a group to avoid potential exclusion or reprisals.

To summarize, the phenomenon of motivated cognition is referred to by different disciplines and terms, and it has slightly different meanings depending on its disciplinary embedding. Often, it is simply referred to as *belief*, although this encompasses information search and processing yielding belief formation or updating.

## Definition and Function of Beliefs

Among the most widely accepted definitions, a belief is a mental state in which a proposition or a statement is held as accurate, frequently without an evaluation (Connors & Halligan, 2015; Schwitzgebel, 2021). Beliefs are thought to have different functions. First, they play an essential role in behavior and decision-making by providing the individual with a basis for understanding and navigating the environment (Halligan & Aylward, 2006). Second, in addition to this informational value, beliefs fulfill psychological and functional needs. They form lenses through which individuals view the world. These lenses are often rose-colored because individuals desire to perceive themselves and others as moral people (Eil & Rao, 2011; Kunda, 1990; Möbius et al., 2022; but for conflicting results, see Ertac, 2011). Updating beliefs about personal vulnerability (e.g., potential entry paths for exploitation) often takes place in a valence-dependent manner, meaning that individuals are likely to embrace good news while tending to neglect bad news (Kuzmanovic & Rigoux, 2017; Moutsiana et al., 2013; Sharot & Garrett, 2016). For example, individuals show a reluctance to update their beliefs about others’ moral character when the incoming information is negative, especially when they have a strong positive prior impression of this person (Kim et al., 2020). Similarly, individuals selectively attach a higher value to positive signals about their own character or ability (Eil & Rao, 2011).

Following newer work in economics, beliefs can even directly enter the utility function of decision makers (Brunnermeier & Parker, 2005; Köszegi, 2006; Loewenstein & Molnar, 2018). This means individuals gain utility not only from the accuracy of a belief but also from its fit with their own psychological needs and motives (Bénabou & Tirole, 2016; Gries et al., 2022; Kunda, 1990). For example, overly optimistic beliefs about one’s own prosociality support a positive self-image. Therefore, subjects may very carefully choose the information they allow to feed into their belief on the basis of anticipating what consequence this would have for their emotions and actions (Golman & Loewenstein, 2018; Sharot & Sunstein, 2020).

## Stability of Beliefs and Potential for Change

Given the benefits that holding certain beliefs are thought to promise for individual decision makers, it comes as no surprise that beliefs are relatively stable and resistant to change. From a developmental perspective, beliefs often originate in biographical experiences (Bellana et al., 2022). For example, individuals are most likely to identify with their parents’ religion (Pew Research Center, 2020), and their perception of fairness is influenced by policies they encountered in their past (Alesina & Angeletos, 2005; Alesina et al., 2001). Further, individuals’ wish for connection with others might often be facilitated through joint and stable beliefs. From a group perspective, this might have been evolutionarily adaptive: Some researchers suggest that sticking to procooperative beliefs, even in light of adversities, serves the public good (and thus the group; Bénabou & Tirole, 2016). Going beyond the role of socialization in forming beliefs, Vellani and colleagues (2022) demonstrated the role of genetic dispositions in beliefs about personal risk, suggesting the inheritability of beliefs.

In line with these findings, cognitive consistency theories assume that core beliefs are stable over time because individuals tend to incorporate new information in a way that they reconcile with their prior beliefs (Festinger, 1957) or produce auxiliary hypotheses that accommodate new information (Kim et al., 2020). Information conflicting with current beliefs is systematically ignored (Golman et al., 2017; Hertwig & Engel, 2016; Sweeny et al., 2010), underweighted (Faia et al., 2022; Kőszegi & Rabin, 2009), discredited (Jain & Maheswaran, 2000), forgotten (Enke et al., 2020; Muller, 2022), or interpreted differently (e.g., Lobeck, 2020; Pettigrew, 1979), whereas confirming evidence blends in effortlessly. The evidence suggests that information relevant to belief formation (and subsequently to decision-making), whether obtained from external sources or memory, can be subject to motivated cognition in two stages: information search and processing.

Abelson (1986) additionally postulated that individuals aim to increase the value of their currently held beliefs by investing in them (e.g., by presenting belief-consistent or belief-defensive behavior). This form of status-quo bias results in the relative consistency of beliefs over time. Lastly, beliefs are often not singular entities but part of an entire narrative needed for sense-making (Molnar & Loewenstein, 2022) and can rarely be changed (or added) without changing a complete set of beliefs. Taken together, this evidence suggests beliefs are relatively stable and may be difficult to change.

However, in many domains, we can observe increasing polarization of opinions (e.g., climate change, vaccination, social-security services; for Gallup analyses, see Dunlap et al., 2016), highlighting that the importance of understanding and changing misguided beliefs is undeniable. Interventions aiming to reduce this polarization must be able to overcome the considerable stability of beliefs over time (Sharot et al., 2022). Importantly, it has been shown that changes in social norms and perceptions of such do occur. For example, in the last 20 years, there has been less stigmatization as acceptance rates of homosexuality have increased in parts of the world (Poushter & Kent, 2020), and beliefs about mental health have improved (Pescosolido et al., 2021). These developments provide evidence that beliefs may change (Akerlof & Dickens, 1982), for instance, as a result of influences from individual experiences, institutional signals, media exposure, or the particular decision context (e.g., Alesina & Angeletos, 2005; Hornik & Niederdeppe, 2008; Jost, 2019; Sharot et al., 2022; Tankard & Paluck, 2016, 2017). For beliefs to change, Bayesian updating assumes that new information must be sufficiently reliable and sufficiently different from the prior (Druckman & McGrath, 2019). Further, Kunda (1990) suggested that individuals can make themselves believe only things that they can make others believe. Thus, changing individuals’ perception of what others find plausible could be one way to overcome belief rigidity.

Changing beliefs does not mean that the previous beliefs are forgotten. Instead, former and current beliefs will often be available in memory for some time, even when the information that was crucial for forming the original belief is proven incorrect (Nisbett & Ross, 1980). Litovsky and colleagues (2022) suggested that individuals might experience discomfort when giving up existing beliefs without gaining equal utility from adopting new ones. For example, the belief that other people are mostly selfish helps to justify their own selfish behavior. Giving up this belief would come at the cost of having to realize that oneself is actually a selfish person or of having to become more prosocial. This may be why individuals often prefer to avoid information that could negatively impact their existing beliefs.

## Processes of Motivated Cognition in the Context of Cooperation

Because (motivated) beliefs are relatively stable and resistant to change but simultaneously crucial for determining one’s cooperation decisions, the processes of their formation and updating are particularly relevant for understanding the cognition-behavior link in the context of cooperation. From the general conceptualization of motivated cognition as a tool to sample and evaluate novel information in the light of prior beliefs, we can derive the function of motivated cognition in the context of cooperation. Theoretical and empirical work shows that preexisting beliefs guide information search and processing. Consequently, individual behavior and social coordination (Ajzen, 1991; Gries et al., 2022) and belief distortions can take place (and may be necessary) at all stages of the cooperative decision-making process (i.e., during information search as well as processing). Integrating the discussed literature, we developed a construct map to systematically display the role of motivated cognition in cooperation (Fig. 1; Gray, 2017).

Visually outlining the concept allows us to display the theoretical contribution of individual studies and the coherence of the overall ideas. Summarizing the evidence in Figure 1 (and the connected empirical evidence summarized in Table 1), the following picture arises: Information available in a specific setting affects cooperation decisions only after being subjected to motivated cognition.

**Table 1.** Overview of Empirical Studies Investigating Relevant Links and Influential Factors of Motivated Beliefs

Topic	Empirical findings
Individual factors	
Moral preferences	Nash et al. (2017); Saccardo & Serra-Garcia (2023)
Political preferences	Braman & Nelson (2007); Caddick & Feist (2021); Ditto et al. (2019); Eichmeier & Stenhouse (2019); Guay & Johnston (2022); Graham & Singh (2023); Jost et al. (2003); Miller et al. (2016); Kahan (2012); Osmundsen et al. (2021); Saunders (2017)
Personality traits	Caddick & Feist (2021); Callan et al. (2014); Coe (2018); Jarvinen & Paulus (2017); Klein & Webster (2000)
Cognitive abilities	Da Silva et al. (2019); Fischer et al. (2022); Habicht et al. (2021); Kahan (2012); Kahan et al. (2012); Knobloch-Westerwick et al. (2020); Pennycook (2022); Pennycook & Rand (2019, 2021); Saunders (2017); Strömbäck et al. (2021); Szebeni et al. (2021)
Situational factors	
Action space	Bardsley (2008); Cappelen et al. (2013); Cullis et al. (2012); List (2007); Zhang & Ortmann (2014)
Task complexity	Caplin et al. (2011); Henckel et al. (2022); Osatuyi et al. (2016); Payne (1976)
Limited cognitive processing (e.g., time pressure)	Bago et al. (2023); De Wit et al. (1989); Strömbäck et al. (2021); Sultan et al. (2022)
Importance	Carlson et al. (2020); Enke et al. (2021); Mayraz (2011); Peterson & Iyengar (2021); Zimmermann (2020)
Information valence	Cosmides et al. (2010); Johnson et al. (2013); Kuzmanovic & Rigoux (2017); Moutsiana et al. (2013); Sharot et al. (2022); Siegel et al. (2018); Skowronski & Carlston (1989)
Norms	Balliet et al. (2014); Kranton & Sanders (2017); Pettigrew (1979)
Uncertainty	Bicchieri et al. (2022); Cetemen et al. (2020); Dana et al. (2007); Exley (2016); Haisley & Weber (2010); Hansson et al. (2021); Rapoport et al. (1992)
Information search: avoidance or exploration	
Internal sampling (e.g., selective memory)	Davidai & Gilovich (2016); Muller (2022); Zimmermann (2020)
External sampling (e.g., filter bubbles)	Barberá (2020); Bar-Tal (2000); Bergh & Lindskog (2019); Epperson & Gerster (2021); Fiedler et al. (2013); Geiß et al. (2021); Golman et al. (2017); Knobloch-Westerwick et al. (2020); Lobeck (2020); Saccardo & Serra-Garcia (2023); Sunstein (2018)
Information processing: belief formation or updating	
Analysis and information-processing effort	Castro Santa et al. (2018); Drobner & Goerg (2022); Kishishita et al. (2023); Schwardmann et al. (2022)
Distorted weighting of information	Fareri et al. (2015); Golman et al. (2016); Kahan (2017); Oprea & Yuksel (2022); Richards & Hewstone (2001); Sharvit et al. (2015); Weisel & Böhm (2015)
Interpretation	Bicchieri et al. (2022); González-Jiménez (2022); O’Leary-Kelly et al. (2017)

Motivated cognition is constructed from the fundamental elements of (biased) information search and processing, resulting in the formation or updating of existing beliefs. Information search pertains to sampling information from internal (e.g., selective memory; Muller, 2022; Zimmermann, 2020) and external (e.g., filter bubbles; Barberá, 2020; Geiß et al., 2021; Sunstein, 2018) information sources. Distortions can enter at this stage when information is sampled selectively, such that unfavorable information is avoided and favorable information is sought out. The collected information is processed through a procedure of analysis, weighting, and interpretation. During this process, certain types of information, such as those that are incongruent or inconvenient, may be given less importance, less in-depth processing, or misinterpreted (and vice versa for favorable information), altering their influence on the ensuing decision (Drobner & Goerg, 2022; Kishishita et al., 2023; Schwardmann et al., 2022). Motivated cognition may be influenced by a variety of individual and situational factors or an interaction thereof. For example, characteristics of the situation may interact with prior beliefs and influence processing (Caddick & Feist, 2021; Enke et al., 2021; Golman & Loewenstein, 2018; Jain & Maheswaran, 2000; Kahan et al., 2012; Strömbäck et al., 2021). The resulting motivated beliefs then feed into decision-making, but might trigger subsequent distorted information search, adding to the cognitive processes.

### Information search: avoidance or exploration

When decision makers face a situation in which they need to decide to cooperate or not, the first step in the process of motivated cognition necessarily relates to information acquisition. Decision makers could sample information from external sources, such as the options available to them, the people involved and affected by potential decisions, and other features of the decision setting. Information from internal sources (e.g., memory, preferences) can also be sampled to determine whether and which (social or personal) norms are relevant for guiding cooperation decisions in a particular instance. Motivated cognition in information search plays out as seeking out and prioritizing certain information, whereas other information may be sought out less or later or avoided entirely.

Research shows that information congruent with prior beliefs or preferences about cooperation is sought out more (Epperson & Gerster, 2021; Fiedler et al., 2013), such that prosocial decision makers seek out information about others’ outcomes more than selfish decision makers, who focus rather on their own outcomes and interaction payoffs instead of contributions. Preferentially sought-out information will consequently play a larger role in decision makers’ process of constructing a choice to cooperate or not. In another example, when confronted with unequal resource distributions, observers could aim to seek out or avoid information about the origin of these disparities, such as whether they arose because of luck or a different effort invested in the task. Motivated cognition could lead decision makers to take a meritocratic point of view, attributing differences to lower effort and thus constructing the belief that no redistributive action against this inequality was necessary (Lobeck, 2020). Individuals search for and attend to information in a manner that reinforces self-enhancing beliefs. For instance, they exhibit a more robust memory for challenges and disadvantages they have surmounted compared with the advantages and privileges they have enjoyed (Davidai & Gilovich, 2016).

Which information is sought out preferentially also determines cooperation with in- and out-group members: Individuals are drawn to (especially positive) information about their in-group (Bergh & Lindskog, 2019) and to information about socially shared beliefs (Bar-Tal, 2000). When facing an in-group member, individuals seek out more information about the decision situation and about their interaction partners’ potential outcomes and consequently cooperate more (Rahal et al., 2020).

Further, there may be bias in the perception of the strength of the relevant social norm. For instance, distorted beliefs assuming a low likelihood of others’ prosocial behavior may result in lower cooperation by the individual and thus function like a self-fulfilling prophecy (Santos et al., 2021). Such pessimistic decision makers may focus on retrieving memories or seeking out external information sources that prove their point, such as examples of others’ uncooperative behavior, and avoid information that could prove them wrong, such as examples of others’ cooperation. The reverse may also be true: Motivated estimations of others’ high trustworthiness in line with one’s motivational states (Baer et al., 2022) can make actors feel safe to cooperate, even when conditions are not optimal (in the context of risk, see Akerlof & Dickens, 1982).

Another perspective on information search in motivated cognition focuses on individuals’ attempts to correct biases within their own decision-making process in an attempt to reduce the impact of motivated beliefs. Saccardo and Serra-Garcia (2023) presented evidence that, whereas some advisors preferred learning about information that could distort their beliefs in a self-serving manner, others preferred limiting the potential for distorted belief formation by avoiding compromising information. This indicates that heterogeneity in preferences about holding motivated beliefs could impact cooperation depending on individual priorities for ethical behavior or avoiding guilt. Therefore, understanding such individual differences is crucial for comprehending the role of motivated cognition in the realm of cooperation.

In sum, which information is sought out or avoided determines how decision makers construct their mental representation of a decision. Biases in information search are one component of motivated cognition in the context of cooperation and have the power to crucially alter the direction of subsequent processing and behavioral steps.

### Information processing: belief updating or formation

Once information has been acquired, it is entered into a processing stage in which it is evaluated and brought into context with other information. Here, motivated cognition may act on the process in terms of assigning excessive weight, devaluing information, or elaborating on the available information in a distorted way. Such distortions influence how beliefs are formed and how they are reevaluated and updated.

#### Information-processing effort

For instance, research shows differences in the analysis and information-processing efforts among decision makers. They tend to invest little time in processing incongruent information, quickly dismissing it as irrelevant while giving favorable information the benefit of the doubt. This triggers a more effortful process of integrating the favorable information into their presently held beliefs (Drobner & Goerg, 2022; Kishishita et al., 2023; Schwardmann et al., 2022). In another example, holding the belief that a partner intends to cooperate boosted individuals’ cooperation responses while triggering differential information processing: Conditional cooperators made cooperative decisions faster, and defection decisions were slowed down among free riders and conditional cooperators because of differences in their own preferences as well as differences in typical beliefs about others’ behavior (Castro Santa et al., 2018).

#### Distorted weighting of information

Information processing often involves distorted weighting of information triggered through belief consonance (Golman et al., 2016; Kahan, 2017), that is, processing to stabilize existing beliefs. For instance, Oprea and Yuksel (2022) found that subjects overweighted others’ beliefs when they were aligned with their motivation while dismissing them when they were not. In the context of in-group bias (Kahan et al., 2012), empirical evidence shows that individuals more rapidly adopt in-group beliefs and disregard or devalue information against these beliefs (Fareri et al., 2015; Richards & Hewstone, 2001). Phenomena such as groupthink (for a review, see Park, 1990) can be conceptualized as distorted information processing and can give rise to discrimination and uncooperative behavior (Sharvit et al., 2015; Weisel & Böhm, 2015).

#### Interpretation

Lastly, acquired information may also be misinterpreted with the goal of forming or retaining desired beliefs. For instance, subjects may misinterpret information about their social environment or about themselves, resulting in beneficial beliefs about their behavior being morally acceptable, their deservingness, or their chances at success (Bicchieri et al., 2022; González-Jiménez, 2022; O’Leary-Kelly et al., 2017). To see themselves in a positive light, individuals attribute failures to external factors such as an uneven playing field or bad luck. In contrast, they credit success to internal attributes such as talent (Zuckerman, 1979).

In sum, how information is processed determines which beliefs are held and, consequently, whether cooperative behavior is put into action. Whether beliefs regard which expectations individuals perceive others to have or the expectations they have of others, or whether beliefs constitute the lens through which social issues and dilemmas are recognized, polarized, or ignored, motivated cognition is the central mechanism that allows us to tell ourselves stories about others and ourselves.

## Influence of Individual Differences on Motivated Cognition

Unsurprisingly, individuals differ in the degree to which they are prone to motivated cognition. There is an array of individual differences potentially implicated in motivated cognition. These include, but are not limited to, moral and political preferences (e.g., political orientation), personality traits (e.g., need for cognitive closure, self-esteem), and cognitive abilities. These variables, either alone or in combination, may shape our mental representation of reality.

### Moral preferences

In an attempt to understand motivated cognition in moral decisions, Nash and colleagues (2017) presented evidence that when moral preferences are heavily focused on the group we are part of, it can be harder for individuals to spot and solve conflicts. This evidence aligns with the observation that, when stepping into the role of an advisor with a conflict of interest, individuals adopt distinct strategies in relation to motivated belief formation. Some individuals first familiarize themselves with their incentives before understanding the potential consequences for their clients. Conversely, others who aim to restrict motivated belief formation first comprehend the consequences for their clients before discovering their personal incentives. This heterogeneity demonstrates how motivated cognition can variably lead to more but also less cooperation, depending on individual preferences for genuine ethicality versus guilt avoidance (Saccardo & Serra-Garcia, 2023).

### Political preferences

A different line of research focuses on the role of political preferences. Although some studies have reported that stronger conservatism is linked to motivated beliefs about political topics (Braman & Nelson, 2007; Caddick & Feist, 2021; Graham & Singh, 2023; Jost et al., 2003; Miller et al., 2016; Osmundsen et al., 2021; Saunders, 2017), others have failed to find such an effect (Ditto et al., 2019; Eichmeier & Stenhouse, 2019; Guay & Johnston, 2022; Kahan, 2012).

### Personality traits

A third individual-difference perspective comes from research on personality traits, such as neuroticism and dispositional anxiety. These traits appear to lower the propensity for motivated cognition (Caddick & Feist, 2021; Jarvinen & Paulus, 2017; but see Coe, 2018) and through this mechanism shape individuals’ beliefs and behavior. Conversely, individuals with a high dispositional need for cognitive closure engaged in less in-depth information processing (Klein & Webster, 2000). Moreover, Callan et al. (2014) presented evidence that self-esteem can influence the extent and form of motivated cognition: Lower self-esteem is related to patterns of self-defeating beliefs and behaviors and to seeking out information that confirms the negative self-view.

### Cognitive abilities

Another line of work has taken a developmental perspective on motivated cognition. Specifically, it has been argued that young children could benefit from motivated cognition because it maintains their motivation while learning despite initial failures (Habicht et al., 2021). This adaptive function of motivated cognition might also explain why some studies have found that higher cognitive abilities go along with a higher, not lower, propensity to display biased belief updating (Da Silva et al., 2019; Kahan, 2012; Kahan et al., 2012; Knobloch-Westerwick et al., 2020; Saunders, 2017; Strömbäck et al., 2021; Szebeni et al., 2021). However, others who tested this link between cognitive abilities and motivated cognition found no evidence of this relationship (Fischer et al., 2022; Pennycook, 2022; Pennycook & Rand, 2019).

In sum, the evidence regarding individual differences and motivated cognition is still sparse, rarely preregistered, and may benefit from reexamination with larger sample sizes (see Schönbrodt & Perugini, 2013). Given the modest number of studies available and the considerable variation in contexts, future research is needed before extrapolation beyond the conclusions of individual studies can be justified.

## Influence of Situational Factors on Motivated Cognition

A substantive amount of research has demonstrated situational influences on cooperative behavior, such as the threat of punishment for noncooperators (Fehr & Gächter, 2000), whether others observe decisions (Wu et al., 2016), what information about potential cooperation partners is available (Bolton et al., 2005), the possibility of communication (Balliet, 2010), and the embedding in a framework of repeated interactions (Van Lange et al., 2011). These situational factors could also affect the prevalence of motivated cognition prior to cooperative choices. For example, receiving threats of sanctions could liberate decision makers to behave selfishly after forming motivated beliefs about the threat issuers’ distrustfulness and uncooperativeness (Houser et al., 2008).

Other research has been more directly devoted to understanding how the situational context determines belief formation and updating (Sharot et al., 2022). In the following sections, we discuss the effects of subjects’ action space, a task’s complexity and importance, information valence, norms, and uncertainty.

### Action space

Decision makers may base their beliefs about which norms exist in a certain task on information about the available options from which they can choose, that is, their action space. For instance, in standard dictator games, dictators can only decide whether and how much to give to the receiver, which may lead them to construct the belief that this game is about giving and that they should signal generosity (Cappelen et al., 2013). When the action set also included the option to take money from the receiver, inviting self-serving beliefs that not taking (or not taking everything) already constitutes socially acceptable behavior, generosity declined (Bardsley, 2008; Cappelen et al., 2013; List, 2007; Zhang & Ortmann, 2014). In the context of taxation, Cullis et al. (2012) showed that different action spaces could influence tax payers’ beliefs about entitlement to income and selfish tax noncompliance: When declaring income to be taxed, tax payers construct beliefs about taxes as perceived losses and are more likely to selfishly evade taxes.

### Mental load

When individuals’ cognitive capacity is occupied or exhausted, hindering in-depth analytical processing, this may affect motivated cognition as well (Lodge & Taber, 2000). One example would be situations with high task complexity, for example, because of a higher number of options to choose from, more attributes defining each alternative (Payne, 1976), or unfamiliarity with the task. Specifically, more complex tasks have been argued to promote a satisficing approach to information search (i.e., reduced information search; see Caplin et al., 2011), possibly impeding accurate belief formation or updating. Although some empirical evidence supports this link between complexity and motivated belief adjustment (Henckel et al., 2022), contrary findings show more belief updating in more complex tasks (e.g., Osatuyi et al., 2016).

The latter matches results of studies using other manipulations for limiting cognitive processing. For example, studies of decisions under time pressure show no change in the propensity for motivated cognition (Bago et al., 2023; Strömbäck et al., 2021; Sultan et al., 2022), and neither does a study using an intervention with sedatives (De Wit et al., 1989). Others have even found evidence for the opposite effect, meaning more reflective modes yielding more bias (Kahan et al., 2012). Together, these results show that the role of cognitive-processing capacity in motivated cognition (and its link with cooperation) remains to be clarified. This should involve scrutiny of whether the manipulations that are used actually target individuals’ cognitive mode or something else. For example, in studies imposing time pressure to limit the availability of cognitive resources, analytical thinking per se might not be hindered. Instead, time pressure might change what an individual’s analytical capacity is used for (e.g., self-deception to evade taxes selfishly or accuracy for monetary reward). Future research should explicitly test the mechanisms through which manipulations of analytical versus intuitive thinking trigger or prevent motivated cognition.

### Task importance

The importance assigned to a task is a further moderator frequently studied for its impact on motivated cognition. Increasing task importance through incentives (Bénabou & Tirole, 2009), and particularly incentivizing accuracy to trigger more analytical processing, has at times led to reduced motivated beliefs (Peterson & Iyengar, 2021; Zimmermann, 2020). Vice-versa, providing incentives to cheat affected motivated cognition in terms of avoidance of inconvenient information (Hochman et al., 2016). However, other studies find minimal effects, even with high stakes (Enke et al., 2021), or no effects at all (Mayraz, 2011). Given these mixed findings, gaining more fine-grained insight into the tradeoff process involved in constructing motivated beliefs is warranted, particularly in the area of cooperation. When even considerable financial incentives do not suffice to sway beliefs toward more accuracy and less motivated cognition, the monetary incentives could be outweighed by anticipated image-damage costs, as in the case of overestimating one’s past generosity to match one’s own fairness standards (Carlson et al., 2020).

### Information valence

The valence of sampled information can trigger motivated cognition. Empirical evidence has frequently demonstrated more belief updating following good rather than bad news (referred to as optimism bias or asymmetric updating), in which the impact of negative information in the belief-updating process is smaller than that of positive information (Moutsiana et al., 2013; Sharot et al., 2011). At the same time, when it comes to forming beliefs about people, decision makers are particularly sensitive to bad news about their interaction partners to avoid being exploited (Cosmides et al., 2010; Johnson et al., 2013). Therefore, it might be reasonable to assume that beliefs about others’ bad character are particularly resistant to change (“once a defector, always a defector”). However, empirical evidence suggests that beliefs held about bad people are more prone to updating than beliefs about good people (Siegel et al., 2018). Holding flexible beliefs about defectors yields a number of advantages (Skowronski & Carlston, 1989), such as opening the door for cooperative behavior in future interactions.

### Norms

Which norms are perceived to be relevant for behavior in a specific decision situation contributes to the emergence of motivated cognition. For instance, in intergroup-decision contexts, which group decision makers identify with and how strongly they feel connected to this group can influence the importance of group-specific norms for subsequent decisions (Kranton & Sanders, 2017). A strong norm prevalent in intergroup-decision contexts is favoring one’s in-group (Balliet et al., 2014). Consequently, facing situations in which cooperation with in- versus out-group members is warranted can give rise to substantive differences in motivated belief formation and updating. In-group-related information may receive a boost in processing, such as when good outcomes from in-group members are attributed to merit instead of luck (but the opposite pattern emerges for out-group members; Pettigrew, 1979). Moreover, when in- and out-groups compete for status, belief consonance can also serve as a status-protection strategy (Sunstein, 2005). When other strong norms exist in parallel, normative conflict may give rise to more motivated cognition. For instance, when group-based in-group favoritism norms collide with overarching fairness norms, individual decision makers can leverage uncertainty about which norm to apply to create room for distorted beliefs and corresponding behavior.

### Uncertainty

Further, research indicates that social and environmental uncertainty forces individuals to adapt and devise strategies to succeed. This adaptation might be achieved through the formation of stronger social bonds, which may explain the increase of motivated beliefs promoting more prosocial behavior under these conditions (Cetemen et al., 2020). In contrast, when individuals are uncertain about others’ actions, they tend to cooperate less (Bicchieri et al., 2022; Rapoport et al., 1992). This type of uncertainty is distinct from social and environmental uncertainty in that it is rooted in mistrust or unpredictability of other individuals’ behaviors rather than the broader environment. Uncertainty about the nature of a task further crucially determines which beliefs are formed about it and whether cooperative behavior follows. For example, in a competitive task, losers who did not receive information about task fairness formed the incorrect belief that the task was stacked against them and selfishly took more from winners compared with losers who had learned that the game was fair (Hansson et al., 2021). Finally, when there is uncertainty about the consequences of one’s actions, such situations can be exploited by individuals to justify self-serving actions. This can be achieved by evading information about the potential outcomes of their actions (Dana et al., 2007) or by interpreting risk (Exley, 2016) and ambiguous information (Haisley & Weber, 2010) about these consequences in a manner that serves their interests.

In sum, although there are some well-documented links between situational differences and motivated cognition, future research consolidating the mechanisms of situational dependence of belief formation and updating, particularly in cooperation, is needed. Promising areas of exploration lie in substantiating findings on the directionality of the effect of task importance and of the effect of environments reducing cognitive capacity.

## Behavioral Consequences: Does Motivated Cognition Block or Boost Cooperation?

The processes of motivated cognition and the influence of both individual and situational factors in the realm of cooperation are complex. This implies that interventions devised to strategically encourage and limit the scope for motivated cognition must be carefully tailored to the respective individuals, and interactions have to be anticipated. Overall, a synthesis of these empirical findings suggests that viewing motivated cognition only as a deficiency or only as an aptitude would be reductionist. Behavioral consequences of motivated cognition in the context of cooperation can be twofold: Either motivated cognition boosts cooperation rates, or it is detrimental for cooperation because it provides room for selfishness or partiality. For instance, individuals may hold stable, inaccurately optimistic beliefs in cooperation situations because they provide social (e.g., social acceptance; Ames, 2004a, 2004b; Kim et al., 2020; Sharot et al., 2022), psychological (Garrett et al., 2014; Korn et al., 2014), and physical health benefits (Garrett et al., 2014; Korn et al., 2014; Strunk et al., 2006; Taylor et al., 2000), or utilities, for the individual. Regardless of its function for the individual, believing in positive outcomes in social interactions is essential for triggering engagement and motivating behavior to reach social goals (Bénabou & Tirole, 2002; Chen & Schildberg-Hörisch, 2019). Such optimistic beliefs might even play a strategic role for individuals with strong social-image concerns (for a similar argument when supporting present-biased agents overcome self-control problems, see Bénabou & Tirole, 2002) or act as a social signal (Burks et al., 2013; Charness et al., 2018; Ewers & Zimmermann, 2015; Schwardmann & van der Weele, 2019). Thus, when individuals form beliefs that reduce social and environmental uncertainty, this may allow for effective coordination and/or support prosocial behavior.

However, although this increased attention toward cooperative action and prosocial behavior produces a helpful short-term optimistic boost, there can be negative side effects associated with it. For instance, a constant mismatch between (overly optimistic) beliefs and observed behavior might trigger disappointment, pessimistic belief adjustment, or even a decoupling of beliefs and cooperative behavior. Furthermore, overly optimistic beliefs can also lead to overcontribution, which results in wastefully depleting resources of prosocials in the long run (Akerlof & Dickens, 1982).

Other negative effects of motivated cognition stem from the specific motivation. If motivated by selfishness, individuals can construct belief systems that make it easier to overlook opportunities for prosocial behaviors or to behave selfishly even when such opportunities are conspicuous. For example, actors may selectively attend to (Dana et al., 2007; Di Tella et al., 2015; Grossman, 2014; but see Serra-Garcia & Szech, 2022) and boost the importance of self-serving arguments, or seek out situational excuses (Exley, 2016; Momsen & Ohndorf, 2020). By constructing positive value for self-serving acts (e.g., as measures of self-care) and constructing negative value for prosocial acts (e.g., assuming ill use of donated funds), individuals can align selfish behavior with a positive self-concept. Such motivated pessimistic beliefs about situations that could afford prosociality may lead to persistent self-centered behavior. Unsurprisingly, this use of motivated cognition is not displayed by all individuals alike but depends on their prosocial disposition: More selfish actors (individualists) are more prone to it. This link is not only found in controlled lab experiments but also has been demonstrated in research on large-scale cooperation problems such as climate-change action (Claessens et al., 2022): Prosocials form beliefs in line with their preferences and are subsequently more willing to take proenvironmental actions, whereas individualists seem to simply evade the social dilemma by refusing to recognize it exists.

Taken together, these results suggest that efficient interventions aimed at improving cooperation have to be tailored to the individual target group. Where motivated cognition can be leveraged to constrain moral wiggle room and boost the importance of social norms, cooperation rates may rise.

## Conclusions

Challenges that require cooperation are complex and increase in scale. The necessity of coordinating numerous behaviors among individuals with different dispositions and in different situational circumstances makes tackling these issues difficult. Previous research has shown that subjects’ engagement in motivated cognition and its effects on cooperation may depend on the context (see Fig. 1 and Table 1) and that it differs among individuals (see Fig. 1 and Table 1). The current state of knowledge will be the start of a fruitful agenda for future research: In addition to gaining a better understanding of when and which individuals are more likely to engage in motivated cognition, it is crucial to investigate the domain specificity of the phenomena (e.g., climate change, health, politics). Moreover, individuals often do not have a single belief or identity related to the complex issues they are facing. Therefore, it is essential to understand which initial beliefs trigger the motivated cognition process for gaining a deeper insight into the mechanisms of motivated cognition. Given the sparse empirical literature on beliefs in the overall context of cooperation, little is known about the tactical construction of individuals’ beliefs to motivate or justify their pro- or antisocial behavior. How does this process differ from rational Bayesian belief updating?

Traditional experiments, although insightful, often miss the mark in unraveling the intricate processes behind choice construction (Berg & Gigerenzer, 2010; Krajbich et al., 2015). To gain a deeper understanding of motivated cognition underpinning cooperation, high-resolution process-tracing methods might serve as a valuable tool. By recording the details of individual information search and processing steps, such investigations can add a new layer of understanding of the mechanisms underlying motivated cognition (Rahal & Fiedler, 2019).

Lastly, understanding in detail the mental steps influenced by motivated beliefs will help inform and distinguish between competing theories of motivated cognition. This could also be a promising starting point for developing tailored interventions to support optimal decision-making in cooperation situations. With the construct map and methodological roadmap provided, we hope that research on motivated beliefs and reasoning will soon systematize the collection of evidence.

## Transparency

*Action Editor:* Henrik Olsson

*Editor:* Interim Editorial Panel
